# Attention‐deficit hyperactivity disorder in children and young persons during the COVID‐19 pandemic. A temporal trends analysis of electronic heath records in Greater Manchester, England

**DOI:** 10.1002/jcv2.70042

**Published:** 2025-09-05

**Authors:** Louise Hussey, Evangelos Kontopantelis, Pearl L. H. Mok, Darren M. Ashcroft, Shruti Garg, Carolyn A. Chew‐Graham, Karina Lovell, Roger T. Webb

**Affiliations:** ^1^ Division of Psychology and Mental Health Manchester Academic Health Sciences Centre The University of Manchester Manchester UK; ^2^ National Institute for Health and Care Research (NIHR) Applied Research Collaboration ‐ Greater Manchester (ARC‐GM) The University of Manchester Manchester UK; ^3^ National Institute for Health and Care Research (NIHR) Manchester Biomedical Research Centre The University of Manchester Manchester UK; ^4^ Division of Informatics, Imaging and Data Sciences University of Manchester Manchester UK; ^5^ Centre for Pharmacoepidemiology & Drug Safety Division of Pharmacy and Optometry School of Health Sciences Manchester Academic Health Sciences Centre The University of Manchester Manchester UK; ^6^ NIHR Greater Manchester Patient Safety Research Collaboration The University of Manchester Manchester UK; ^7^ Royal Manchester Children's Hospital Manchester University NHS Foundation Trust Manchester UK; ^8^ Faculty of Medicine and Health Sciences School of Medicine Keele University Newcastle‐under‐Lyme UK; ^9^ Division of Nursing, Midwifery & Social Work University of Manchester Greater Manchester Mental Health NHS Foundation Trust Manchester UK; ^10^ Mental Health Nursing Research Unit Greater Manchester Mental Health NHS Foundation Trust Manchester UK

**Keywords:** ADHD, children and young people, COVID‐19, deprivation, ethnicity

## Abstract

**Background:**

The incidence of attention‐deficit hyperactivity disorder (ADHD) in children and young people has increased in recent years. Disease frequency varies according to sociodemographic characteristics. There are seasonal patterns in ADHD diagnosis and prescribing with rates falling during school holidays. COVID‐19 societal restrictions may have exacerbated ADHD symptoms. Electronic health records were utilised to examine temporal trends throughout the pandemic in the diagnosis and treatment of ADHD by ethnicity and social deprivation in Greater Manchester, England.

**Methods:**

We conducted a time‐series analysis of all diagnosed episodes of ADHD and associated medication prescribing among patients aged 1–24 years using the Greater Manchester Care Record (GMCR). The 60‐month observation period was split into four temporal phases: Pre‐pandemic (1/2019–2/2020); Pandemic Phase 1 (3/2020–6/2021); Pandemic Phase 2 (7/2021–12/2022) and Post‐Pandemic (1/2023–12/2023). Rate ratios by sex, age, ethnicity, and neighbourhood‐level Indices of Multiple Deprivation (IMD) quintile were modelled using negative binomial regression.

**Results:**

Overall, ADHD incidence and medication prescribing rates increased throughout the study period. Rates of increase were much higher in females than in males. Particularly large increases in ADHD incidence and medication prescribing were observed in Asian females, with post‐pandemic incidence rates being seven times higher compared to the Pre‐Pandemic phase. In addition, the ADHD medication prescribing rate was 90% higher for Asian females than for White females. Results showed a large increase in incidence and prescribing rates in the least deprived group, particularly in males where incidence rates increased by 83% compared to the most deprived quintile.

**Conclusions:**

ADHD incidence and prescribing rates differ between sociodemographic groups, plausibly due to cultural and behavioural differences in the way ADHD symptoms are presented or perceived. It is therefore important that there is greater understanding of how different demographic subgroups exhibit ADHD behaviour to help ensure timely diagnosis and access to the required support.

## Key Points

**What's known?**

- The incidence of attention‐deficit hyperactivity disorder (ADHD) has increased in recent years.
- COVID‐19 societal restrictions may have exacerbated ADHD symptoms.

**What's new?**

- Using electronic health records, we observed increases in ADHD incidence and medication prescribing rates throughout the 60‐month study period particularly in females.
- The largest increases in incidence and prescribing rates were observed in Asian females and also in individuals living within the least deprived neighbourhoods.

**What's relevant?**

- Cultural and behavioural factors may influence the way ADHD symptoms are presented and perceived. Greater understanding of these issues is needed to ensure timely diagnosis and access to the required support.

## INTRODUCTION

The incidence of attention‐deficit hyperactivity disorder (ADHD) has increased in recent years. This is now one of the most common neurodevelopmental conditions diagnosed in childhood with an estimated global prevalence of 5% (NIHR, 2023). A recent UK study in adults estimated a 20‐fold increase in diagnoses and a 50‐fold increase in ADHD medication prescriptions from 2000 to 2018 (McKechnie et al., 2023). The increase in ADHD‐related prescriptions has been highlighted recently due to a shortage in ADHD medication, attributed to a combination of manufacturing issues and an increase in global demand (ADHD UK, 2024b; Lewis & Khong, 2024; New Scientist, 2023). In the UK in 2015/2016, 107,155 individuals received an ADHD prescription, and by 2022/2023 this had more than doubled to 277,640 (ADHD UK, 2023; NHS Business Service Authority, 2023).

Rates of ADHD vary according to sociodemographic characteristics. Incidence of diagnosis and prescribing rates are three to five times greater in males than in females (Hire et al., 2018; Mac Avin et al., 2020; McKechnie et al., 2023). Evidence has also shown a disparity by socioeconomic position (McKechnie et al., 2023; G. Russell et al., 2014) including a systematic review examining 42 studies which estimated that children (aged 5–19) in families of lower socioeconomic position were approximately twice as likely to have ADHD than those in families of higher socioeconomic position (A. E. Russell et al., 2016). Socioeconomic inequality in prescribing has also been highlighted in a study based in Scotland. Despite higher incidence rates of ADHD, children from least affluent backgrounds were less likely to have received medication post‐diagnosis than those from a higher socioeconomic position (Pearce et al., 2024), Conversely, there is little published evidence regarding UK ADHD incidence or diagnostic rates specific to ethnic groups (Gulati, 2021; G. Russell et al., 2014). However, a review published in 2020 synthesised the (largely US‐based) evidence pertaining to ADHD care for ethnic minority children. A notable disparity between African American compared to Caucasian children and young persons (CYP) was found, which was attributed to issues including cultural factors and problem recognition, attitudes of teachers and differential access to mental health services (Slobodin & Masalha, 2020).

Symptoms of ADHD include inattention, hyperactivity, and impulsivity. If left undiagnosed and untreated, these symptoms can negatively impact child wellbeing and future development. ADHD is associated with lower educational attainment and can impact upon interpersonal relationships and daily functioning (ADHD UK, 2024a; May et al., 2021). ADHD symptoms can cause significant disruption and impairment in educational activities. As such, a diagnosis can often involve intervention through staff noticing problematic behaviours with rates falling during holidays (ADHD UK, 2024c; Cohen et al., 2016; Efron, 2017; Shyu et al., 2016) and closures of educational establishments evidently impacted during the COVID‐19 pandemic (Bannett et al., 2022; Bilu et al., 2023). Societal restrictions (‘lockdowns’) implemented during the COVID‐19 pandemic were associated with symptom exacerbation among those diagnosed with ADHD (Freedman et al., 2023; Shah et al., 2021). In studies examining the impact of the pandemic on a range of mental health problems in CYP, conditions such as anxiety and depression increased, whilst rates of ADHD diagnoses decreased. The authors have attributed this to closures of schools and other educational establishments and the lack of intervention from staff (Freedman et al., 2023; Shah et al., 2021). Little evidence was found of the differential impact of the COVID‐19 pandemic on rates of ADHD diagnosis by age, gender and socioeconomic status (Zemer et al., 2024). However, Marmot et al. have described how the pandemic amplified inequalities and it has been suggested that financial difficulties during this time negatively impacted on children's mental health (Marmot & Allen, 2020; Moulin et al., 2023). A survey of UK paediatricians reported that health service disruption also impacted on the frequency of ADHD diagnosis (NHS Digital, 2020b). Almost half of the respondents ceased to offer new ADHD CYP assessments, and only 5% were able to conduct the physical monitoring usually carried out for individuals diagnosed prior to suggesting medication (Ogundele et al., 2022).

In the UK, COVID‐19 pandemic societal restrictions included school closures and remote learning from 18 March 2020 until the start of the Autumn term in September 2020, and again during January and February 2021 (Brown & Kirk‐Wade, 2021; Timmins, n.d.). In addition to the closure of educational establishments, the whole of the UK population experienced societal restrictions of varying stringency between March 2020 and July 2021 (Brown & Kirk‐Wade, 2021). Greater Manchester (GM) is a large conurbation in the North‐West region of England with approximately 2.8 million residents. The GM population experienced some of the most stringent and prolonged measures nationally (Brown & Kirk‐Wade, 2021). Compared to other UK regions, this metropolitan area has relatively high levels of deprivation and is more ethnically diverse (Visit North West, 2023).

Studies using electronic health records to examine mental and physical health conditions have shown a significant decrease in incidence rates during the early stage of the pandemic (Carr et al., 2021; Hussey et al., 2024; Steeg et al., 2021; Trafford et al., 2023). In this study we aimed to utilise GM‐wide digital records to examine how incidence rates for diagnosed ADHD and related medication prescribing in CYP changed during different stages of the COVID‐19 pandemic. This enabled us to investigate whether ADHD incidence rates also fell within the early stage of the pandemic or if they continued to rise as has been reported previously (NIHR, 2023; McKechnie et al., 2023). These temporal patterns were examined separately by sex, ethnicity and deprivation.

## MATERIALS AND METHODS

### Data source access approval

We utilised data extracted from the Greater Manchester Care Record (GMCR). The GMCR, established in 2019, holds conurbation‐wide electronic health records, including approximately three million patient records from 443 general practices (Health Innovation Manchester, 2024). To enable access to anonymised data pertaining to individual patients, a research protocol (reference RQ‐051) was submitted and approved in accordance with the national Control of Patient Information (COPI) notice (NHS Digital, 2020a).

### Delineation of the study cohort

To conduct a time‐series analysis we examined monthly totals of all ADHD clinical codes denoting incident cases of ADHD among patients aged 1–24 years from 1 January 2019 (first date available) to 31 December 2023, a 60‐month (5 year) observation period. We included individuals aged 1–24 years to capture the full developmental spectrum of ADHD diagnosis and treatment, encompassing early childhood, school age, adolescence, and young adulthood. Incident (as opposed to prevalent) cases were identified by examining all previous entries in a patient's electronic health record to ensure that the counts included were the first reports of ADHD. In addition, we obtained information on all ADHD medications prescribed. These included medications classified as: Dexamfetamine Sulphate, Atomoxetine hydrochloride, Guanfacine hydrochloride, Lisdexamfetamine dimesylate, Methylphenidate hydrochloride, Modafinil, and Pitolisant hydrochloride (Connelly, 2023). The incidence and prescription monthly counts were stratified by sex, age, ethnicity (White, Black and Asian) and area‐level deprivation as measured by Index of Multiple Deprivation (IMD) 2019 quintiles. The IMD is a measure of deprivation at small area level across England, based on seven domains: Income; Employment; Education, Skills & Training; Health & Disability; Crime; Barriers to Housing & Services; and Living Environment (Department for Levelling Up, Housing and Communities, 2020). Incidence and prescribing rates were calculated by dividing the numerator data by monthly estimates of population denominators using three data sources: GMCR; Census 2021 (Office for National Statistics, 2023); and NHS Digital GP practice lists (NHS Digital, 2023). Monthly GP practice size information was extracted from NHS Digital. Census 2021 information was used to validate the proportional distribution by sex, age, ethnicity and IMD, which was then applied to the GM population.

To enable examination of changes in incidence and prescribing rates throughout and beyond the COVID‐19 pandemic, rates during three time periods since the start of the pandemic (from March 2020) were compared with the preceding pre‐pandemic period as follows: *Pre‐pandemic*: January 2019‐February 2020 (months 1–14); *Pandemic Phase 1* (societal restrictions in place): March 2020 to June 2021 (months 15–30); *Pandemic Phase 2* (all restrictions lifted): July 2021 to December 2022 (months 31–48) and *Post‐Pandemic*: January to December 2023 (months 49–60).

We decided to call the fourth phase ‘Post‐Pandemic’ whilst acknowledging that the SARS‐CoV‐2 virus (which can result in COVID‐19 disease) continued to be transmitted within the population. A survey of immunologists and other communicative disease researchers showed that 90% of respondents believed that the coronavirus will become endemic (Phillips, 2021). In the UK, the last time the COVID‐19 booster was offered to persons aged 50 and older was in the Winter of 2022. After this the vaccine roll‐out was restricted to those aged 75 and over plus other vulnerable groups (BBC News, 2023). It was therefore considered reasonable to delineate this last 12 months of the study period as a separate epoch.

### Statistical analyses

The numbers of incident diagnoses of ADHD and medication prescription counts were aggregated to give monthly totals and then subsequently grouped into the four time periods described above. Monthly incident and prescribing rates and rate ratios (and their 95% confidence intervals) were estimated using negative binomial regression. A variable for calendar month (1–12) was fitted in the model to adjust for seasonality. Rate ratios were estimated to compare rates during Pandemic Phases 1 and 2 and the Post‐Pandemic phase with the Pre‐Pandemic period as the reference category. In addition, rate ratios specific to sociodemographic groups were calculated by fitting an interaction term between the categorical demographic group of interest and the time‐period. These were: sex, age group, ethnicity and IMD quintile. Evidence (ADHD UK, 2024c; Efron, 2017) has shown that educational establishments play a key role in ADHD diagnosis. Therefore, a binary variable was generated and used in separate analyses to discern any impact on rates due to the GM‐wide closure of educational establishments across GM during lockdown measures (March 2020 to August 2020 and January 2021 to February 2021) or summer break (August in every year of the observation period). Analyses were performed in Stata v17.

## RESULTS

### Incidence and prescribing rates across the whole study period

Over the 60‐month observation period, the study dataset held monthly counts totalling 11,846 incident cases of ADHD and 261,876 prescription items issued for ADHD medication (Table 1). The incidence rate of ADHD diagnosis was 70% higher in males than in females and the prescribing rate was approximately 3.5 times higher (Table 1 and Table S1). ADHD diagnoses were recorded in all ages (from as young as 1‐year old) in both males and females. However, overall, males were diagnosed at a younger age than females. Incidence rates were highest amongst 6 to 12‐year‐olds for males whereas, for females, rates were highest in those aged 13–19. Both males and females were observed to have the highest prescribing rates in the 13–16 years age group. Incidence and prescribing rates were much higher for individuals of White ethnicity compared to the Black and Asian ethnic groups. ADHD medication prescribing rates were generally found to be highest in the most deprived neighbourhoods. Overall, there was no discernible pattern observed in incidence across the IMD quintiles. Our analysis showed that the diagnosis of ADHD increased significantly by 42% when the schools were open compared to when they were closed (incidence rate ratio, IRR 1.42; 95% CI: 1.20–1.71). There was, however, no significant difference in prescribing rate related to school closure (Figure S1).

**TABLE 1:** Population denominators, numerator values and diagnosed incidence for attention‐deficit hyperactivity disorder (ADHD) and prescribing rates of ADHD medication per 100,000 person‐months.

	Mean population denominator per month	Incidence					
	*N* (%)	ADHD			Medication		
		*N*	Mean per month	Rate per 100,000 person‐months	*N*	Mean per month	Rate per 100,000 person‐months
Gender							
Males	478,680 (50.9)	7533	125.6	26	204,661	3411.0	714
Females	461,883 (49.1)	4313	71.9	15	57,215	953.6	205
Total	940,563 (100.0)	11,846	197.4	‐	261,876	4364.6	‐
Ethnicity							
Males							
White	316,627 (66.1)	5755	95.9	30	165,448	2757.5	870
Black	31,759 (6.6)	123	2.1	7	2689	44.8	141
Asian	87,419 (18.3)	272	4.5	5	4574	76.2	87
Other	42,875 (9.0)	1030	17.2	‐	24,275	404.6	‐
Missing data	0 (0)	353	5.9	‐	7675	127.9	‐
Females							
White	305,322 (66.1)	3356	55.9	18	48,433	807.2	264
Black	30,651 (6.6)	89	1.5	5	616	10.3	33
Asian	84,627 (18.3)	171	2.9	3	1020	17.0	20
Other	412,283 (8.9)	536	8.9	‐	4731	78.9	‐
Missing data	0 (0)	161	2.7	‐	2415	40.3	‐
Age group							
Males							
1–5	94,118 (19.7)	89	1.5	2	12	0.2	0.2
6–9	82,920 (17.3)	2375	39.6	48	21,109	351.8	424
10–12	63,245 (13.2)	1828	30.5	48	56,047	934.1	1479
13–16	79,600 (16.6)	1706	28.4	36	75,519	1258.7	1587
17–19	55,956 (11.7)	638	10.6	19	28,136	468.9	840
20–24	102,547 (21.4)	897	15.0	15	23,838	379.3	388
Females							
1–5	89,707 (19.3)	28	0.5	0.5	31	0.5	1
6–9	79,239 (17.1)	760	12.7	16	4625	77.1	97
10–12	60,294 (13.0)	568	9.5	16	11,704	195.1	323
13–16	75,284 (16.2)	994	16.6	22	17,759	296.0	393
17–19	53,588 (11.5)	756	12.6	23	10,486	174.8	324
20–24	105,880 (22.8)	1207	20.1	19	12,610	210.0	198
IMD^a^							
Males							
1	216,107 (45.1)	3394	56.6	26	104,630	1743.8	807
2	93,733 (19.6)	1525	25.4	27	40,035	667.3	711
3	55,134 (11.5)	1009	16.8	30	23,384	389.7	706
4	57,256 (12.0)	839	14.0	24	20,677	344.6	601
5	49,832 (10.4)	759	12.7	25	15,871	264.5	530
Missing data	6618 (1.4)	7	0.1	‐	64	1.1	‐
Females							
1	202,322 (43.8)	1785	29.8	15	26,803	446.7	220
2	90,095 (19.5)	991	16.5	18	11,723	195.4	216
3	53,374 (11.6)	579	9.7	18	7196	119.9	224
4	53,785 (11.6)	455	7.6	14	5488	91.5	169
5	46,771 (10.1)	502	8.4	18	5987	99.8	212
Missing data	15,536 (3.4)	1	<0.1	‐	18	0.3	‐

^a^ Neighbourhood‐level Index of Multiple Deprivation (IMD): 1 = most deprived, IMD 5 = least deprived.

### Sex‐specific temporal trends

**FIGURE 1:**
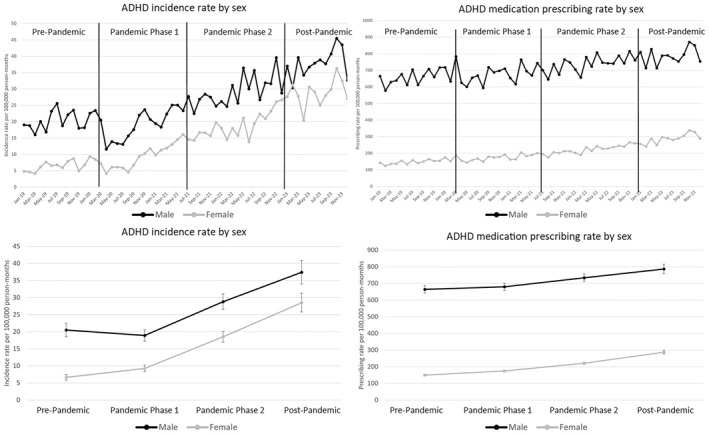
Monthly sex‐specific incidence rates per 100,000 person‐months and temporal trends in rate ratios (vs. the pre‐pandemic period, 95% CI) stratified by sex for ADHD diagnoses and for ADHD medication prescribing. ADHD, attention‐deficit hyperactivity disorder.

**FIGURE 2:**
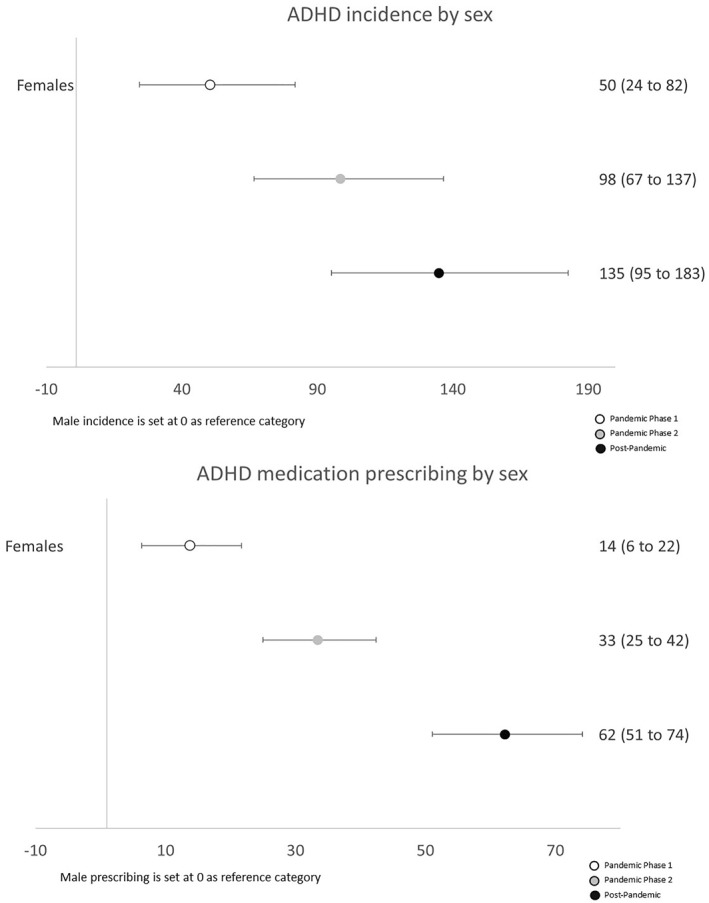
Relative percentage increases or decreases in rate ratios (95% CIs) for females (vs. male children and young persons) during Pandemic Phases 1 and 2 and Post‐Pandemic: attention‐deficit hyperactivity disorder incidence.

**FIGURE 3:**
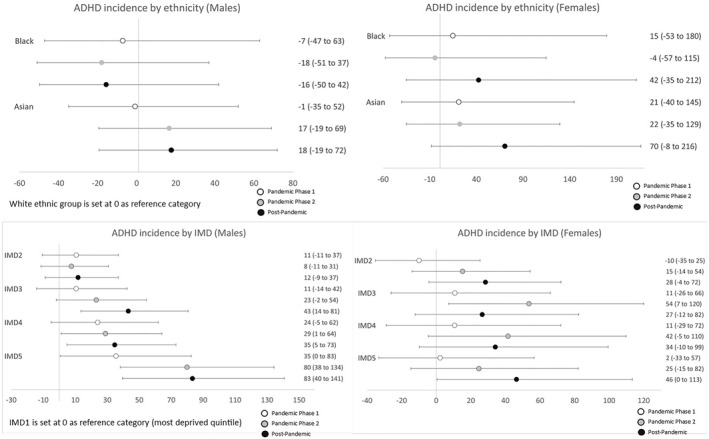
Relative percentage increases or decreases in rate ratios (95% CIs) for minority ethnic groups (vs. White children and young persons) and for IMD quintiles 2–5 (vs. the most deprived quintile, IMD1) during Pandemic Phases 1 and 2 and Post‐Pandemic: attention‐deficit hyperactivity disorder incidence. IMD, Index of Multiple Deprivation.

**FIGURE 4:**
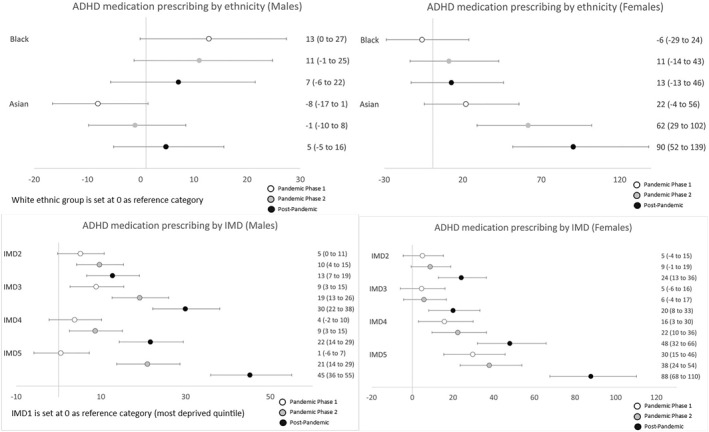
Relative percentage increases or decreases in rate ratios (95% CIs) for minority ethnic groups (vs. White children and young persons) and for IMD quintiles 2–5 (vs. the most deprived quintile, IMD1) during Pandemic Phases 1 and 2 and Post‐Pandemic: attention‐deficit hyperactivity disorder medication prescribing. IMD, Index of Multiple Deprivation.

In males, there was a slight (non‐significant) decrease in ADHD incidence rates during the first phase of the pandemic. However, overall, incidence and medication prescribing rates increased continuously throughout the 60‐month study period for both males and females, particularly in the Pandemic Phase 2 and Post‐Pandemic periods (Figure 1). Sex‐specific rate ratios for the three phases of the pandemic compared to Pre‐Pandemic rates are presented in Table 2. During the Post‐Pandemic period, incidence rates in males had increased by 82% (1.82; 95% CI: 1.60–2.08) and the rate in females was four times higher than it was pre‐pandemic (4.31; 95% CI: 3.62–5.13). Prescribing rates increased by 18% (1.18; 95% CI: 1.13–1.24) in males and by 92% (1.92; 95% CI: 1.80–2.04) in females. These analyses are illustrated in Figures 2, 3, 4 where the ratios are expressed as a percentage increase or decrease compared to the respective reference categories.

**TABLE 2:** Temporal trends in rate ratios (95% CIs) versus the pre‐pandemic period, (95% CI) stratified by sex—incidence of attention‐deficit hyperactivity disorder (ADHD) and ADHD medication prescribed.

ADHD incidence	Pandemic Phase 1	Pandemic Phase 2	Post‐Pandemic
Males	0.92 (0.81–1.05)	1.40 (1.24–1.59)	1.82 (1.60–2.08)
Females	1.40 (1.17–1.68)	2.80 (2.37–3.31)	4.31 (3.62–5.13)

ADHD medication	Pandemic Phase 1	Pandemic Phase 2	Post‐Pandemic
Males	1.02 (0.98–1.07)	1.11 (1.06–1.16)	1.18 (1.13–1.24)
Females	1.16 (1.09–1.24)	1.47 (1.38–1.56)	1.92 (1.80–2.04)

Figure 2 shows how the percentage increases in incidence and prescribing rates are much higher in females versus males. In the final Post‐Pandemic phase, the ADHD incidence rate in females increased by 135% (95% CI: 92%–188%) and ADHD medication prescribing increased by 62% (95% CI: 51%–74%) compared to males.

### Temporal trends by ethnicity

Sex‐specific rate ratios for ethnicity (and for IMD) comparing rates during the pandemic phases with pre‐pandemic levels are presented in Table 3. The differences between these rate ratios are expressed as percentage values in Figures 3 and 4, with the White ethnic group set to 0. Rates per 100,000 person‐months for all four study time periods are illustrated graphically in Figures S2–S5. Males of all ethnic groups showed a fall in incidence rate in Pandemic Phase 1 followed by increased rates throughout the remaining study period. For males of White ethnicity, Post‐Pandemic rates were 86% higher than they were Pre‐Pandemic (1.86; 95% CI: 1.67–2.06) and for Asian males this increase was more than twice Pre‐Pandemic rates (2.32; 95% CI: 1.61–3.35). The increases observed in Black males was not statistically significant. However, for this ethnic group there was a significant increase in prescribing rates throughout the observation period with Post‐Pandemic rates 28% (1.28; 95% CI: 1.14–1.44) higher than Pre‐Pandemic rates. Among females, for those of White ethnicity, Post‐Pandemic rates were almost four times higher than they were Pre‐Pandemic (3.97; 95% CI: 3.33–4.74); for Black females this increase was almost six times Pre‐Pandemic rates (5.96; 95% CI: 2.78–12.77) and for Asian females approximately seven times higher (6.93; 3.84–12.49). The increase in rates in Black and Asian females was greater than the increase in White females although this was not shown to be significant (Figure 3). There was, however, a significant difference between ethnic groups with regards to ADHD medication prescribing in females. Those of Asian ethnicity had increased rates of prescribing that was 62% (95% CI: 29%–102%) higher than the increase in White females in Pandemic Phase 2 and 90% (95% CI: 52%–139%) higher in the Post Pandemic period (Figure 4).

**TABLE 3:** Temporal trends in sex‐specific rate ratios (95%) versus the pre‐pandemic period by ethnic group and IMD quintile—incidence of attention‐deficit hyperactivity disorder (ADHD) and ADHD medication prescribed.

	ADHD incidence			ADHD medication		
	Pandemic Phase 1	Pandemic Phase 2	Post‐Pandemic	Pandemic phase 1	Pandemic Phase 2	Post‐Pandemic
Ethnicity						
Males						
White	0.92 (0.83–1.03)	1.39 (1.26–1.54)	1.86 (1.67–2.06)	1.02 (0.99–1.06)	1.11 (1.07–1.15)	1.19 (1.15–1.23)
Black	0.83 (0.47–1.45)	1.16 (0.69–1.93)	1.55 (0.93–2.60)	1.17 (1.04–1.31)	1.23 (1.10–1.38)	1.28 (1.14–1.44)
Asian	0.98 (0.64–1.48)	1.80 (1.25–2.60)	2.32 (1.61–3.35)	0.94 (0.86–1.03)	1.10 (1.01–1.20)	1.25 (1.14–1.36)
Females						
White	1.34 (1.12–1.61)	2.67 (2.26–3.16)	3.97 (3.33–4.74)	1.18 (1.11–1.25)	1.45 (1.37–1.54)	1.89 (1.77–2.01)
Black	1.71 (0.71–4.11)	2.69 (1.21–5.98)	5.96 (2.78–12.77)	1.08 (0.83–1.41)	1.56 (1.22–1.99)	2.09 (1.63–2.67)
Asian	1.72 (0.87–3.39)	3.29 (1.79–6.06)	6.93 (3.84–12.49)	1.42 (1.12–1.79)	2.36 (1.90–2.92)	3.59 (2.90–4.43)
IMD^a^						
Males						
IMD1	0.85 (0.75–0.98)	1.25 (1.10–1.41)	1.57 (1.38–1.79)	1.00 (0.97–1.04)	1.05 (1.01–1.08)	1.07 (1.03–1.11)
IMD2	0.95 (0.81–1.31)	1.34 (1.15–1.56)	1.76 (1.50–2.05)	1.05 (1.01–1.10)	1.15 (1.10–1.20)	1.21 (1.15–1.26)
IMD3	0.95 (0.76–1.19)	1.55 (1.26–1.89)	2.26 (1.86–2.75)	1.09 (1.04–1.14)	1.25 (1.19–1.30)	1.39 (1.32–1.46)
IMD4	1.05 (0.83–1.33)	1.63 (1.32–2.03)	2.12 (1.70–2.64)	1.04 (0.99–1.10)	1.14 (1.08–1.19)	1.30 (1.24–1.37)
IMD5	1.17 (0.89–1.52)	2.27 (1.79–2.87)	2.90 (2.29–3.67)	1.00 (0.94–1.07)	1.27 (1.20–1.34)	1.55 (1.46–1.65)
Females						
IMD1	1.40 (1.15–1.71)	2.41 (2.01–2.89)	3.69 (3.07–4.43)	1.10 (1.04–1.17)	1.36 (1.29–1.44)	1.62 (1.52–1.72)
IMD2	1.28 (0.99–1.67)	2.83 (2.25–3.56)	4.79 (3.83–5.98)	1.16 (1.07–1.25)	1.49 (1.38–1.60)	2.01 (1.86–2.17)
IMD3	1.57 (1.09–2.25)	3.67 (2.66–5.06)	4.67 (3.37–6.47)	1.15 (1.05–1.26)	1.43 (1.32–1.56)	1.94 (1.78–2.11)
IMD4	1.50 (1.00–2.25)	3.41 (2.37–4.89)	4.89 (3.40–7.01)	1.27 (1.16–1.40)	1.66 (1.52–1.81)	2.39 (2.18–2.61)
IMD5	1.40 (0.96–2.04)	2.95 (2.11–4.12)	5.33 (3.84–7.35)	1.45 (1.30–1.62)	1.88 (1.70–2.09)	3.05 (2.75–3.39)

^a^Neighbourhood‐level Index of Multiple Deprivation (IMD): 1 = most deprived, IMD 5 = least deprived.

### Temporal trends by Index of Multiple Deprivation quintiles

The results for the temporal trends in IMD have been shown alongside those for ethnicity (Table 3, Figures S2 and S3 and in Figures 3 and 4 with the most deprived quintile (IMD1) set as the reference category). The initial slight decrease in incidence rates in males was only observed in the three highest quintiles of deprivation (IMD1 to IMD3). Other than these groups, there was an increase in ADHD incidence and ADHD medication prescribing rates shown for both males and females in all IMD quintiles throughout the study period. This increase was greatest in the least deprived group (IMD5). Compared to the Pre‐Pandemic period, Post‐Pandemic ADHD incidence rates in males were almost three times higher (2.90; 95% CI: 2.29–3.67) and in females over five times higher (5.33; 95% CI: 3.84–7.35). In addition, Post‐Pandemic medication prescribing rates in males were 55% higher (1.55; 95% CI: 1.46–1.65) and in females over three times higher (3.05; 95% CI: 2.75–3.39). Pre‐Pandemic, ADHD incidence rates in males and females were highest in the most deprived group (IMD1). However, although still increasing, rates in this quintile become the lowest compared to the less deprived quintiles. In males, the incidence rate amongst the least deprived (IMD5) increased by 80% (95% CI: 37%–136%) and 83% (95% CI: 38%–143%) in last two study periods compared to IMD1. In females, a comparative increase of 46% (95% CI: 0%–115%) in the most recent study phase was observed. Female ADHD medication prescribing rates showed a similar pattern. The least deprived quintile changed from being one of the lowest prescribing rates to the highest Post‐Pandemic. However, in males, prescribing rates remained the highest in the most deprived quintile throughout the study period. For both males and females, compared to the most deprived quintile, prescribing increased for the least deprived quintile significantly, particularly in the Post‐Pandemic period (males by 45%; 95% CI: 36%–55% and in females by 88%; 95% CI: 67%–111%).

We conducted further analysis stratifying by both IMD quintile and ethnicity by classifying ethnicity into two very broad groups that is White and non‐White (a more granular stratification could not be implemented as it would have resulted in very small numerator counts). These rates (per 100,000 person‐months) are presented in the supplementary materials (Figures S4 and S5). For those of White ethnicity, the most deprived quintile had the highest incidence and medication prescribing rates. Conversely, in the those of non‐White ethnicity, the most deprived quintile had the lowest rates.

## DISCUSSION

### Summary of main findings

Analysis of electronic health records from the GMCR database has enabled us to examine rates of ADHD among CYP aged 1–24 years within a population of approximately 2.8 million people over a 5‐year period, included the COVID‐19 pandemic. Overall, ADHD incidence and medication prescribing rates increased throughout the 60‐month study period. These increases were much greater in females than in males. ADHD incidence and medication prescribing showed increases in rates across the study period for all ethnic groups, particularly in Asian females. Analysis by area‐level deprivation indicated that there was a particularly large increase observed for the least deprived quintile. Pre‐Pandemic, incidence rates were highest in the most deprived quintile. However, although still increasing, during the Post‐Pandemic phase rates in this group become the lowest compared to least deprivation quintiles. For both males and females, compared to the most deprived quintile, prescribing increased significantly for those least deprived, particularly during the post‐pandemic period.

### Comparison of findings with other published evidence

#### Incidence and prescribing rates across the whole study period

It is widely established that incidence rates of ADHD and associated prescribing are much higher in males than in females (Kok et al., 2020; Mac Avin et al., 2020; McKechnie et al., 2023; Renoux et al., 2016). Incidence and prescribing rates were lowest in the 1–5 years age group. Our study included individuals from 1 year of age as evidence has shown that hyperactivity and inattention can often be noticeable before the age of two with parents reporting behaviour/temperament concerns as early as 12 months of age (Askeland et al., 2022; Miller et al., 2021). Male CYP were diagnosed with ADHD at a younger age than females, as was reported from a study examining healthcare records in Wales (mean age of 10.9 in boys vs. 12.6 in girls). This suggested that ADHD in females is often misdiagnosed or superseded by a diagnosis of other conditions such as anxiety and depression (Martin et al., 2024). Girls may present with sub‐threshold symptoms or inattention and boys are more likely to exhibit disruptive behaviour such as hyperactivity or impulsivity (Grevet et al., 2006; Quinn & Madhoo, 2014). Similar to other studies, our results show that incidence and prescribing rates are highest in CYP of White ethnicity compared to Black and Asian ethnic groups (Evans et al., 2004; Shi et al., 2021; Singh, 2012; Slobodin & Masalha, 2020). In our study, an incident case record required an ADHD diagnosis to have been entered into a patient's electronic health record. Therefore, it is difficult to discern the degree to which observed differences by ethnicity (and other sociodemographic factors) reflect actual difference in incidence or a disparity in seeking and accessing healthcare for the condition. There are also cultural factors and the interpretation of children's behaviour by parents, caregivers and teachers to consider. A survey of parent‐reported ADHD in the USA showed CYP of Black ethnicity were least likely to have been diagnosed with and medicated for ADHD despite having more symptoms associated with the condition. The authors suggest that cultural factors may influence the perceptions of these behaviours (Coker et al., 2016). Furthermore, clinicians may be more responsive to parents of White CYP seeking help with a possible ADHD diagnosis than parents of other ethnicities. For example, between 2004 and 2006, Black students aged 10–12 years were more likely than those of White ethnicity to exhibit ADHD symptoms (12% vs. 7%) but were less likely to have been clinically diagnosed (9% vs. 14%) (Abdelnour et al., 2022; Coker et al., 2016).

Similar to our findings, there is strong evidence that increased rates of ADHD incidence and prescribing is associated with lower socioeconomic position (Hire et al., 2018; McKechnie et al., 2023; Nunn et al., 2020; A. E. Russell et al., 2016). A study based on UK primary care data estimated that rates of new ADHD diagnosis and first prescriptions were approximately 2–3 times higher in the most deprived versus the least deprived quintile (McKechnie et al., 2023). An examination of CYP with an ADHD diagnosis in Sheffield, England found that for every incremental decrease in area‐level deprivation (according to IMD decile) there was a 10% decrease in the odds of receiving ADHD medication (Nunn et al., 2020). Higher levels of deprivation may have an association with increased rates of ADHD incidence and prescribing due to greater exposure to detrimental family and school environments. However, conversely, it has been shown that families experiencing challenging psychosocial circumstances are less likely to seek help and teachers and healthcare professional more likely to attribute behavioural difficulties to inadequate parenting than to a diagnosis of ADHD (Nunn et al., 2020). When the analyses were run separately for White and non‐White CYP, there was a clear demonstration of increased rates with higher levels of deprivation in those of White ethnicity. However, ADHD incidence and medication prescribing rates within the most deprived neighbourhoods in non‐White CYP were lower than those from all other IMD quintiles. This may indicate that the mediation of reduced help‐seeking and service utilisation has a greater impact on ethnic minorities living in areas of higher deprivation.

#### Temporal trends over the 5‐year study period

A previous study carried out using similar methodology examining trends in anxiety disorders and depression showed a decrease in rates of in the early stages of the pandemic (Hussey et al., 2024). This sharp decrease, also demonstrated in other studies (Carr et al., 2021; Steeg et al., 2021; Williams et al., 2020), likely arose due to the abrupt and profound change in GP accessibility that occurred during this period. Apart from an initial small non‐significant decrease in males, our results showed an increase in ADHD incidence and prescribing rates throughout the study period. Surveys have shown there were increases in ADHD‐related symptoms due to a change in people's routines and structures (Davoody et al., 2022; Kavoor & Mitra, 2021; Ogundele et al., 2022; Shah et al., 2021). However, this increase is likely to have been mediated by service restrictions. Paediatricians reported major disruptions to ADHD services with almost half ceasing the assessment of new ADHD patients and greater usage of telemedicine for ADHD diagnostic appointments (Ogundele et al., 2022). Our study also shows that there was a 42% increase in ADHD incidence when schools were open. Therefore, closure of schools and colleges during lockdowns, as well as concurrent disruption to ADHD diagnostic services, are likely to have mediated the observed increases in the frequency of recorded ADHD diagnoses in patients' electronic health records.

The increase in rate of ADHD incidence and prescribing was higher for females compared to males. ADHD research has been biased towards males with observed outcomes then generalised to girls. Symptoms of ADHD can exhibit differently in females, including less externalisation of behaviours, and masking of gender atypical differences, which has resulted in underdiagnosis. Latterly, wider recognition of these factors has likely led to these increased rates (Littman & Wagenberg, 2023; Mowlem et al., 2019).

#### Temporal trends by ethnicity and Index of Multiple Deprivation quintiles

In the Post‐Pandemic phase of our study, the increase in ADHD incidence rates and associated medication prescribed was higher in Asian females compared to White females. A study based in New Zealand examined ADHD medication dispensing to CYP between 2007 and 2017 (D’Souza et al., 2020). Similar to our findings, the Asian ethnic group had highest rate of increase over time (of 190%). In addition, particularly at the end of the study period, this ethnic group showed the highest prevalence in the least deprived quintile, suggesting that the rise in ADHD dispensing prevalence may be driven by those living in the least deprived neighbourhoods. Chan et al. compared questionnaires rating 18 ADHD symptoms from parents in the UK with those in Hong Kong. Hong Kong parents had significantly lower levels of parental tolerance regarding their children's problematic behaviour (i.e., conformity and self‐control). These cultural differences meant that many more children were categorised as being within the clinical range for an ADHD diagnosis (Chan et al., 2022).

Increases in the incidence of ADHD diagnoses and prescribed medication are driven by multiple factors (McKechnie et al., 2023) including increased awareness amongst patients, parents and healthcare professionals, and changing societal attitudes. We found that these increases were particularly large in the least deprived quintile. Research has shown that individuals within higher socioeconomic groups have better health outcomes and interactions with healthcare services. This includes longer GP consultation times and a higher proportion of referral for diagnostic tests and specialist care (Allen et al., 2021; Cookson et al., 2016; Furler et al., 2002). It is possible that the increased ADHD awareness may have less of an impact on those living in more deprived neighbourhoods. Furthermore, this increased awareness has led to greater demand on NHS resources and long waiting lists. As such, many people are seeking an ADHD diagnosis privately; an option only available to families that can afford it (Gudka et al., 2024).

### Strengths and limitations

GMCR data includes individual‐level ethnicity and neighbourhood‐level social deprivation, enabling the examination of ADHD trends among these strata. The GMCR was introduced to improve patient care by making information available to front‐line workers across the health and care services. Therefore, although data are primarily from general practice, conditions diagnosed elsewhere can also be recorded. These analyses were conducted over a relatively recent 5‐year period enabling an observation of post‐pandemic trends to the end of 2023 calendar year. At the time of writing, few comparable studies including this recent timeframe had been published. The GM population is relatively ethnically diverse and deprived, which may mean that our findings are less generalisable to other more affluent and less diverse areas. During the COVID‐19 pandemic, GM experienced particularly restrictive measures, which may also limit the study's external validity. The GMCR dataset consists of electronic health records and thus an ADHD diagnosis through an interaction with a healthcare provider is required for an incidence case to be ascertainable. The extent to which an observed increase in incidence is due to an actual change that has occurred in the population or to a change in health seeking behaviour is unknown. It is also unknown how the COVID‐19 pandemic or the greater awareness of ADHD have impacted upon GPs' prescribing behaviour. Remote ADHD diagnosis was increasingly used during the pandemic and CYP with suspected ADHD were prescribed medication without a physical examination. This may lead to different outcomes than an in‐person consultation (Ogundele et al., 2022). Furthermore, we do not know whether there were any changes in prescription duration or dosage. Our 60‐month observation period was grouped into four phases and the division of months into these four phases may be considered arbitrary. However, the authors based this pragmatically on notable changes in societal restrictions. We were unable to use optimal interrupted time series analysis due to insufficient pre‐pandemic data (which started in January 2019) to calculate predicted frequencies based on longitudinal trends. We restricted our analysis by ethnicity to three groups (White, Black and Asian) due to limitations in numbers and resulting statistical power. In addition, analysis of an ‘Other’ ethic group combines a wide range of ethnicities and therefore has limits in interpretation when discussing the possible impacts of cultural issues. The ethnicity data were subject to some missing information. Watkinson et al. assessed the impact on missing ethnicity information in a study using the same GMCR data to examine vaccination rates during the COVID‐19 pandemic. Their analysis concluded that it did not substantially bias results (Watkinson et al., 2022).

## CONCLUSIONS

It is unknown whether the rise in ADHD diagnosis and associated prescribing was due to an increase in incidence or to changes in health seeking behaviour during the COVID‐19 pandemic and beyond. However, it illustrates a substantial rise in demand for healthcare provision to support those affected. The results of this study have shown how ADHD and prescribing rates differ between sociodemographic groups. An examination of the literature has highlighted how ADHD is underdiagnosed within certain groups due to an absence of typical ADHD behaviour. The recent rise in diagnosis in females has accompanied greater awareness of the condition, likely due to increased understanding that symptoms may present differently compared to those exhibited by males. Similarly, increases have been seen in some ethnic groups whereby cultural issues may impact on how ADHD behaviours are manifested or perceived. It is of upmost importance that there is greater understanding of how different demographic subgroups exhibit ADHD behaviour and ensure that individuals are diagnosed without delay to thereby enable them to access the support needed in a timely manner. Furthermore, it is also essential to understand barriers that exist in accessing appropriate support with a view to reducing health inequalities.

## AUTHOR CONTRIBUTIONS

Roger T. Webb, Louise Hussey and Evangelos Kontopantelis conceived and designed the study and developed the statistical analysis plan. Louise Hussey conducted the statistical analysis and authored the paper. Roger T. Webb supervised the study and production of this article. All authors contributed to the interpretation of the findings and subsequent edits of the manuscript.

## CONFLICT OF INTEREST STATEMENT

The authors declare no conflicts of interest.

## ETHICAL CONSIDERATION

This study involves human participants but ethical approval by an Ethics Committee or Institutional Board was not required. A research protocol (RQ‐051 Webb) was approved enabling access to data pertaining to individual patients in accordance with the national Control of Patient Information (COPI) notice.

## Supporting information

Supplementary Material

## Data Availability

Data subject to third party restrictions‐Data from the Greater Manchester Care Record was made available to researchers as part of the response to the COVID‐19 pandemic. In accordance with the national Control of Patient Information (COPI) notice, access to the data pertaining to individual patients was subject to the approval of a research protocol and not publicly available.
