# Supplementary material for: Attention‐deficit hyperactivity disorder in children and young persons during the COVID‐19 pandemic. A temporal trends analysis of electronic heath records in Greater Manchester, England
Source: JCPP Adv. 2025 Sep 5;6(2):e70042. doi: 10.1002/jcv2.70042 (PMC13260708; doi:10.1002/jcv2.70042)
Supplement: Supplementary file 1 — Supplementary Material [file JCV2-6-e70042-s001.pdf]

Supplementary Materials

Table S1. Incidence rate ratios (95% CI) for incidence/prescribing rates compared to reference categories for ADHD and related medication stratified by sex, age group, ethnic group and Index of Multiple Deprivation (IMD) quintile

	ADHD Incidence	ADHD Medication
**Gender**		
Males (Ref)	1	1
Females	0.59 (0.50-0.70)	0.29 (0.27-0.31)
**Ethnicity**		
Males		
White (Ref)	1	1
Black	0.21 (0.17-0.26)	0.16 (0.15-0.17)
Asian	0.17 (0.15-0.20)	0.10 (0.10-0.10)
Females		
White (Ref)	1	1
Black	0.27 (0.20-0.36)	0.13 (0.11-0.14)
Asian	0.18 (0.14-0.24)	0.08 (0.07-0.09)
**Age Group**		
Males		
1 to 5 (Ref)	1	1
6 to 9	30.34 (23.81-38.65)	2000.08 (1132.56-3532.11)
10 to 12	30.56 (23.96-38.97)	6975.20 (3950.16-12316.85)
13 to 16	22.64 (17.75-28.88)	7483.62 (4238.15-13214.41)
17 to 19	12.04 (9.37-15.48)	3959.78 (2242.34-6992.61)
20 to 24	9.25 (7.22-11.85)	1827.81 (1035.03-3227.82)
Females		
1 to 5 (Ref)	1	1
6 to 9	30.79 (19.99-47.43)	169.19 (117.09-244.48))
10 to 12	30.05 (19.47-46.38)	562.48 (389.54-812.19)
13 to 16	41.76 (27.15-64.25)	683.66 (473.54-987.03)
17 to 19	44.64 (28.98-68.77)	563.81 (390.44-814.16)
20 to 24	36.36 (23.65-55.89)	344.20 (238.38-497.00)
**IMD***		
Males		
IMD1 (Ref)	1	1
IMD2	1.04 (0.91-1.19)	0.88 (0.84-0.92)
IMD3	1.16 (1.01-1.34)	0.88 (0.84-0.92)
IMD4	0.93 (0.81-1.08)	0.75 (0.71-0.78)
IMD 5	0.97 (0.84-1.12)	0.66 (0.63-0.69)
Females		
IMD1 (Ref)	1	1
IMD2	1.25 (0.99-1.57)	0.98 (0.88-1.09)
IMD3	1.23 (0.97-1.56)	1.02 (0.91-1.13)
IMD4	0.96 (0.76-1.22)	0.77 (0.69-0.86)
IMD5	1.22 (0.96-1.54)	0.96 (0.86-1.07)

**Neighbourhood-level Index of Multiple Deprivation (IMD): 1=Most deprived, IMD 5=Least deprived*

Figure S1: Relative percentage (95% CI) increases or decreases in rate ratios for the total population over the 60-month study period when schools were open (vs school closures). ADHD incidence and ADHD medication prescribing.

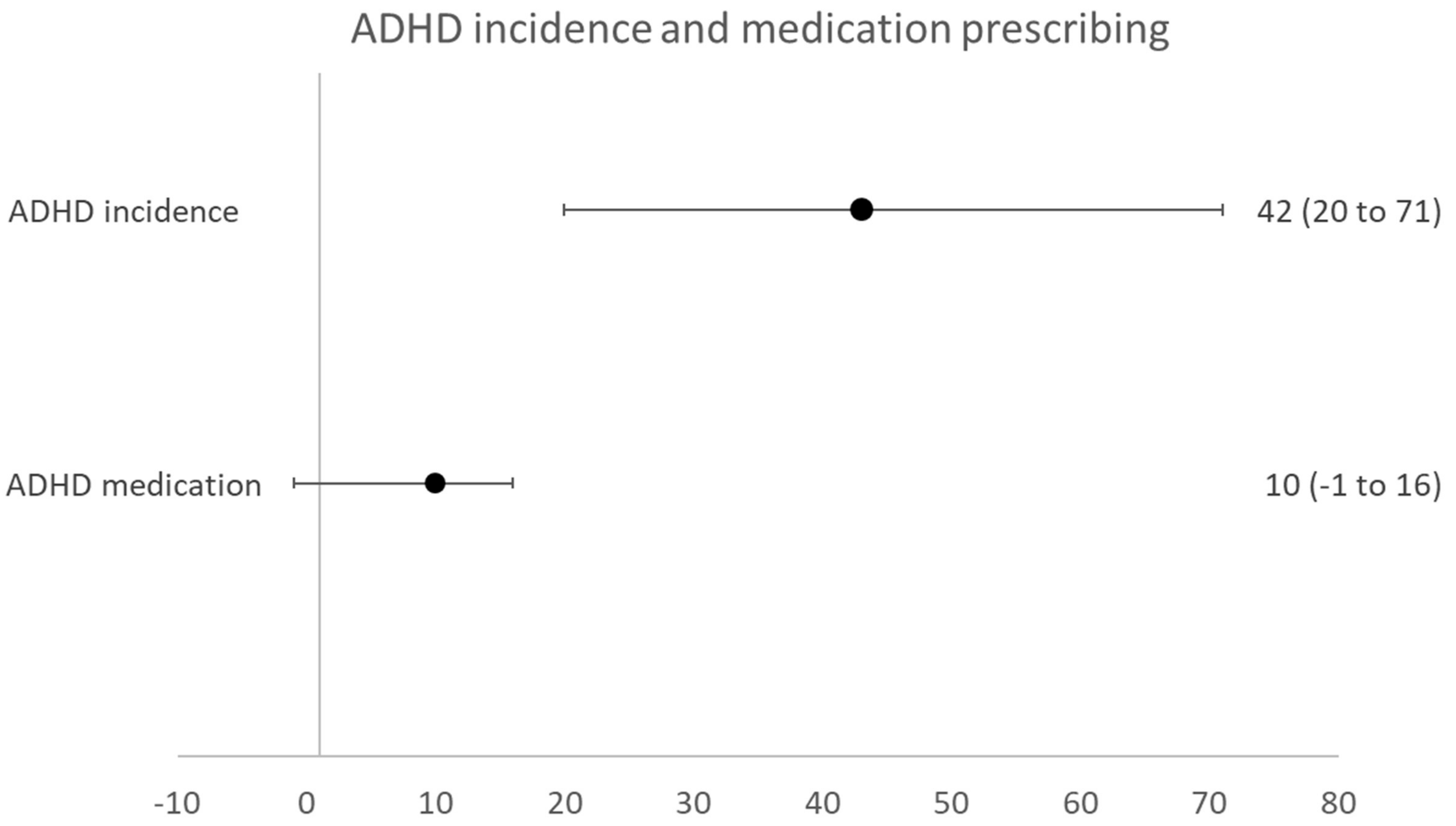

Figure S2: Temporal trends in sex-specific ADHD incidence rates per 100,000 person-months (and 95% CI) stratified by ethnic group and IMD quintile

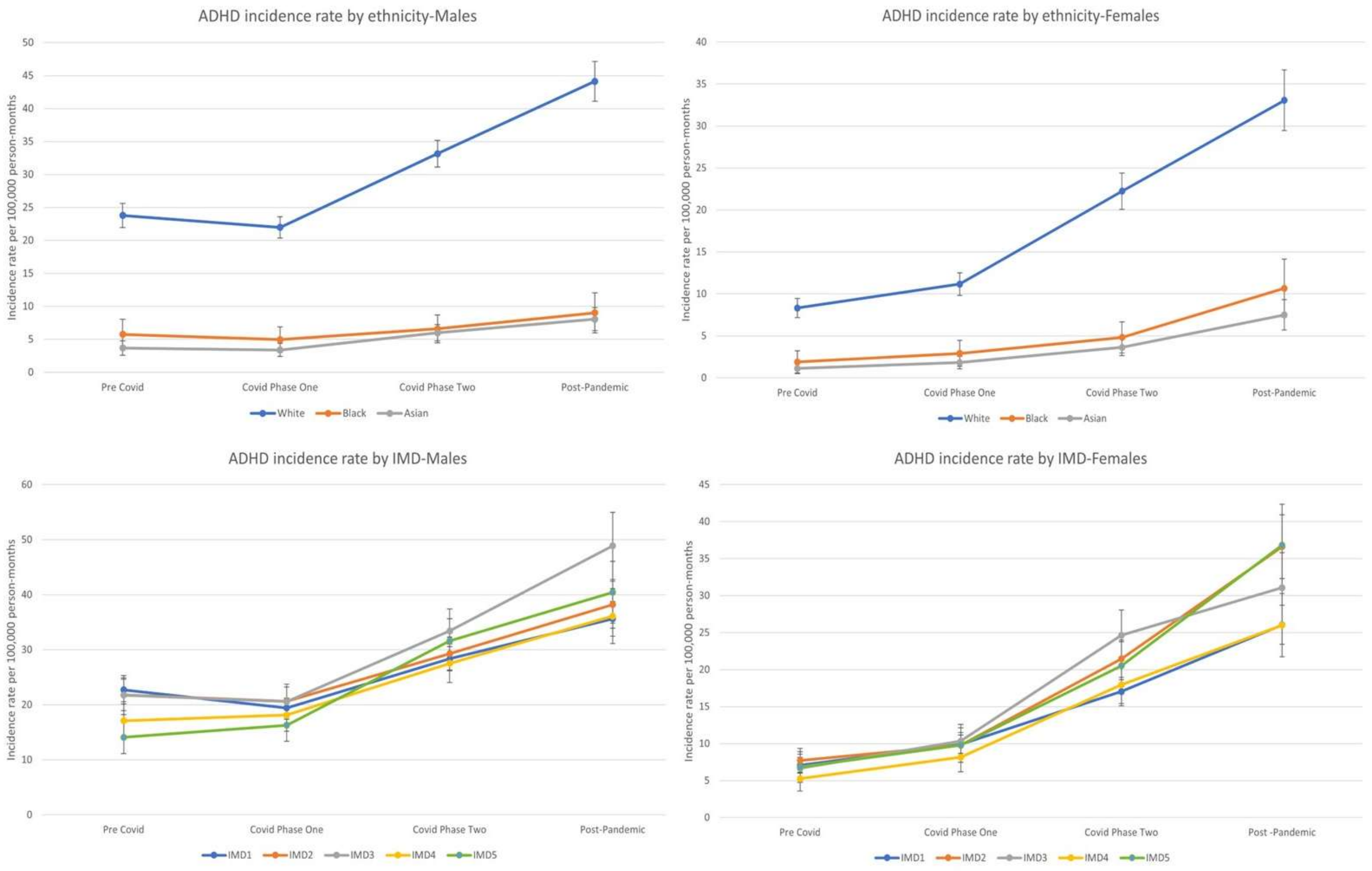

**Neighbourhood-level Index of Multiple Deprivation (IMD): 1=Most deprived, IMD 5=Least deprived*

Figure S3: Temporal trends in sex-specific ADHD medication prescribing rates per 100,000 person-months (and 95% CI) stratified by ethnic group and IMD quintile

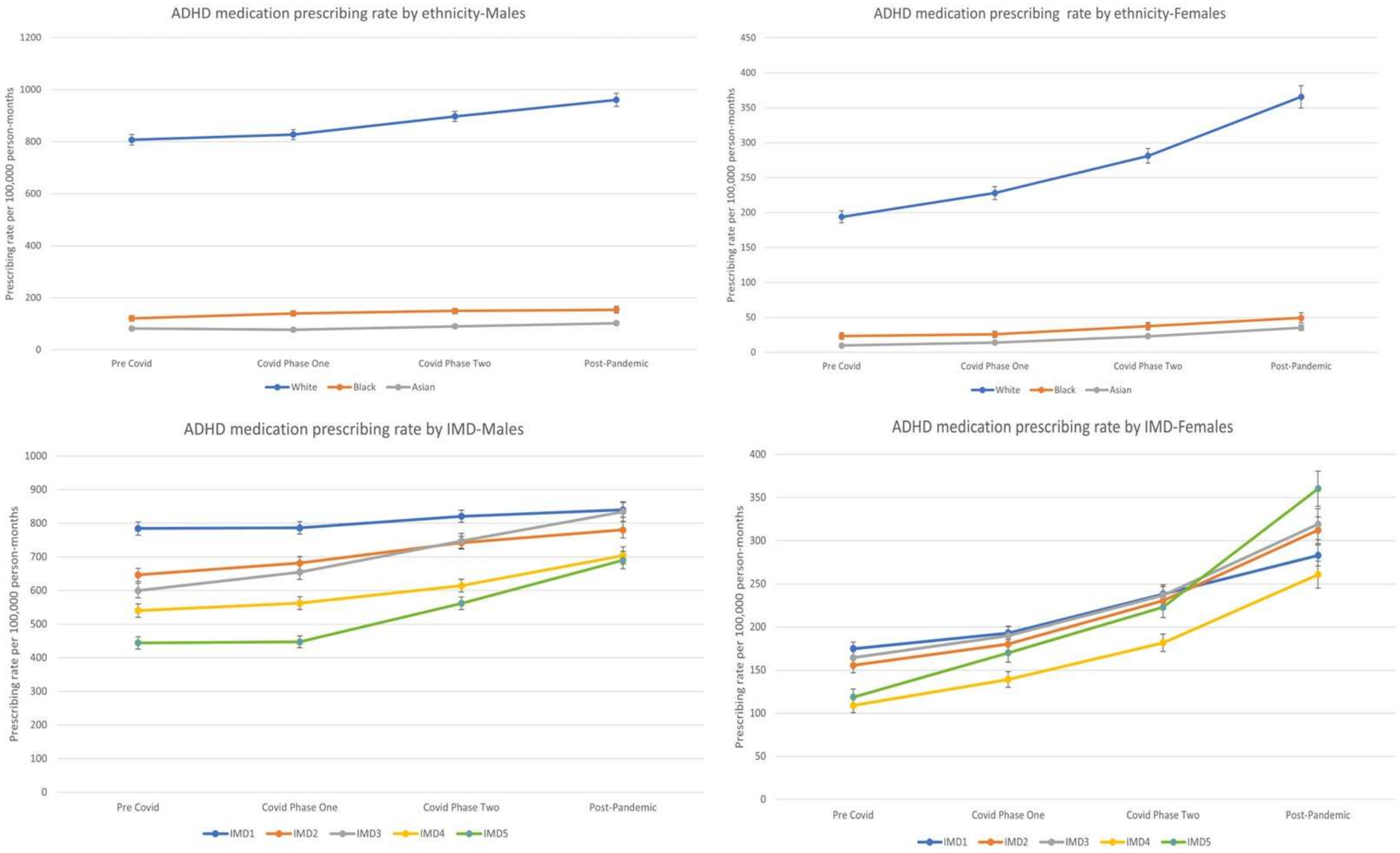

**Neighbourhood-level Index of Multiple Deprivation (IMD): 1=Most deprived, IMD 5=Least deprived*

Figure S4: Temporal trends in sex-specific ADHD incidence rates per 100,000 person-months (and 95% CI) stratified by IMD quintile for White and Non-White ethnic groups

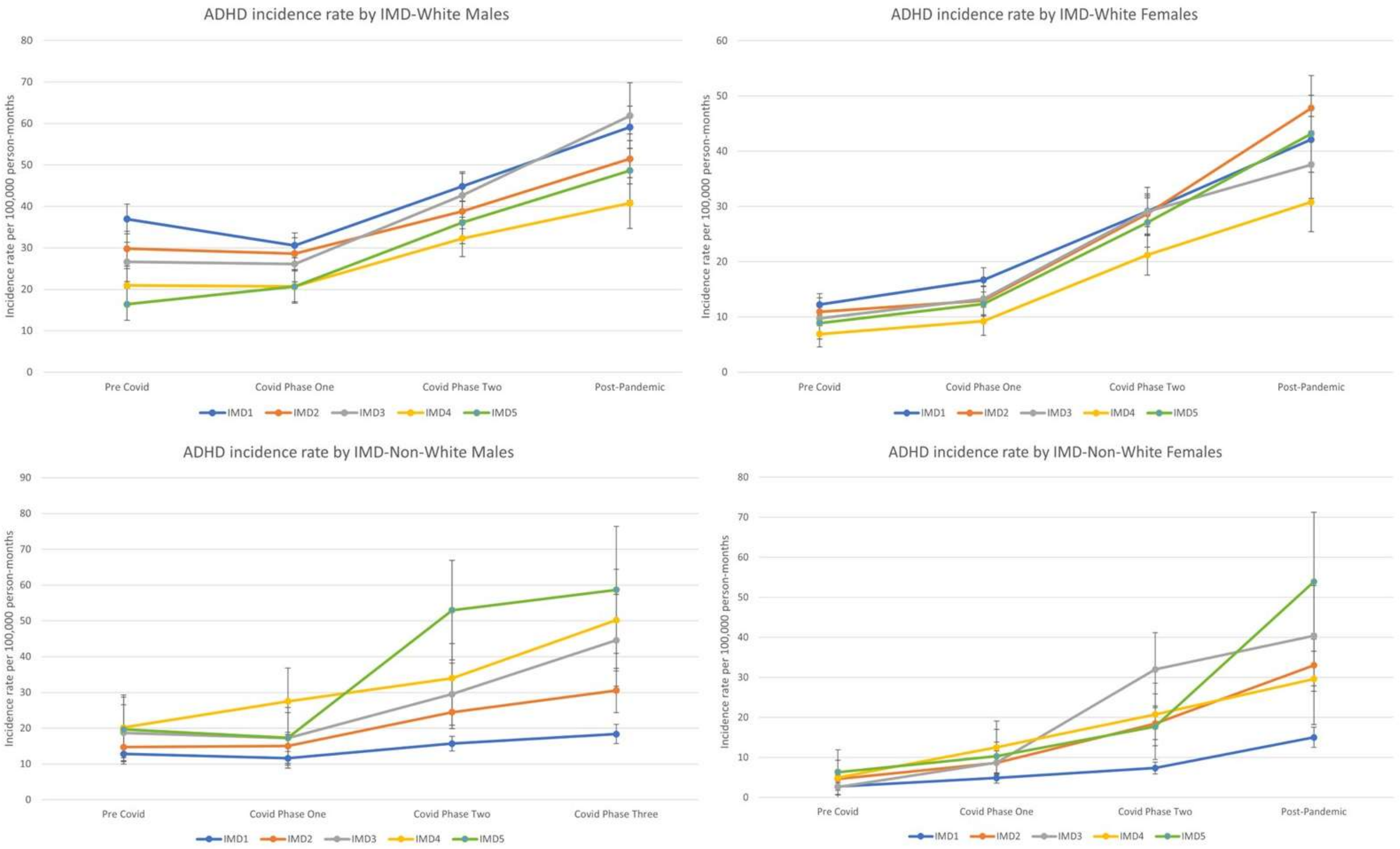

**Neighbourhood-level Index of Multiple Deprivation (IMD): 1=Most deprived, IMD 5=Least deprived*

Figure S5: Temporal trends in sex-specific ADHD medication prescribing rates per 100,000 person-months (and 95% CI) stratified by IMD quintile for White and Non-White ethnic groups

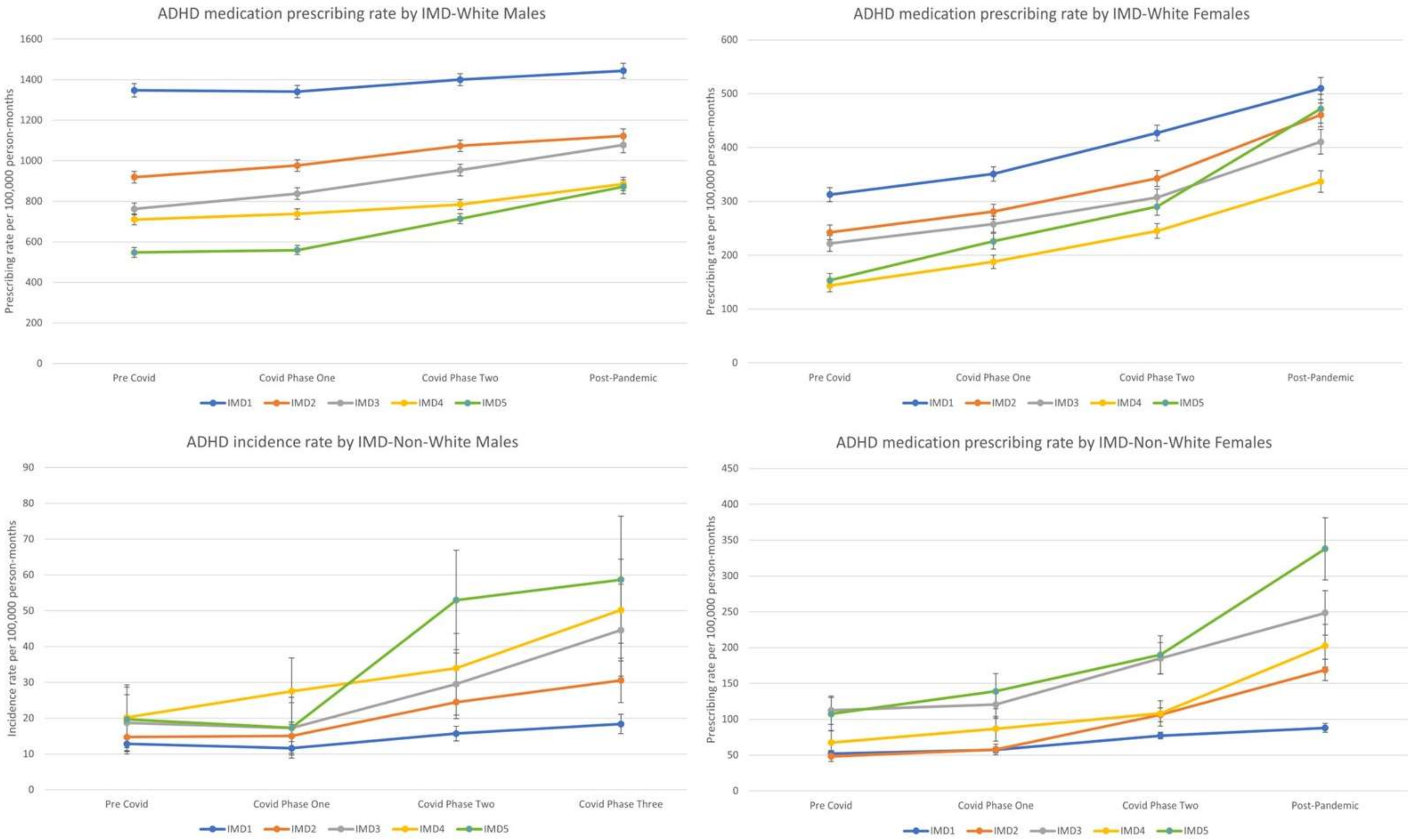

**Neighbourhood-level Index of Multiple Deprivation (IMD): 1=Most deprived, IMD 5=Least deprived*
